# Assessing the palatability of different meats consumed in a biodiversity hotspot to inform alternative protein interventions

**DOI:** 10.1111/cobi.70026

**Published:** 2025-04-22

**Authors:** Charles A. Emogor, Isa B. Ebri, Benedict A. Atsu, Dominic S. Ogu, Omini B. Iferi, Andrew Balmford

**Affiliations:** ^1^ Conservation Science Group, Department of Zoology University of Cambridge Cambridge UK; ^2^ Wildlife Conservation Society Calabar Nigeria; ^3^ Pangolin Protection Network (Pangolino) Calabar Nigeria

**Keywords:** alternative protein projects, conservation intervention, consumer preference, meat palatability, pangolin, Pholidota, wild meat, carne silvestre, intervención de conservación, pangolín, Pholidota, preferencias del consumidor, proyecto de proteína alternativa, sabor de la carne

## Abstract

Alternative protein interventions are common in conservation. They aim to reduce the hunting or consumption of wildlife by promoting substitutes. However, selecting suitable meat substitutes is challenging because many factors drive wild meat consumption. Palatability, one such factor, drives consumer food preference and is potentially crucial in determining meat substitutability in the context of alternative protein interventions. Nonetheless, there have been few assessments of wild meat palatability compared with other options. We collected data on the meat palatability of 96 animal species via a standardized questionnaire administered to 570 hunters, household members, and wild meat vendors (190 respondents in each group) in southeast Nigeria to examine the potential for wild meat substitution. We found positive correlations in the palatability of different species across pairs of respondent groups, highlighting preference similarities. We did not find a statistically significant difference in the average palatability of domestic meat, fish, invertebrates, or wild meat, suggesting scope for substitution based on palatability. Among mammalian orders, ungulates, carnivores, primates, and rodents had similar palatability, but pangolins (*Phataginus* sp. and *Smutsia gigantea*) had higher palatability than all orders except rodents. These findings suggest that substituting wild meat with other types of meat based on palatability might be appropriate, except for pangolins, which can only be suitably substituted with rodents.

## Abstract (es)

Análisis del sabor de diferentes carnes consumidas en un punto caliente de biodiversidad para informar las intervenciones de proteínas alternativas

**Resumen:**

Las intervenciones de proteínas alternativas son habituales en la conservación. Su objetivo es reducir la caza o el consumo de animales silvestres mediante sustitutos. Sin embargo, la selección de sustitutos cárnicos adecuados es un reto porque son muchos los factores que impulsan el consumo de carne silvestre. Uno de ellos es el sabor, que determina las preferencias alimentarias de los consumidores y puede ser crucial para determinar con qué se puede sustituir la carne en el contexto de las intervenciones de proteínas alternativas. Sin embargo, se han realizado pocos análisis del sabor de la carne silvestre en comparación con otras opciones. Recopilamos datos sobre el sabor de la carne de 96 especies animales mediante un cuestionario estandarizado para 570 cazadores, miembros de hogares y vendedores de carne silvestre (190 encuestados en cada grupo) en el sureste de Nigeria para analizar el potencial de sustitución de la carne silvestre. Encontramos correlaciones positivas en el sabor de las distintas especies entre pares de grupos de encuestados, lo que resalta las similitudes en las preferencias. No encontramos diferencias estadísticamente significativas en el sabor medio de la carne doméstica, el pescado, los invertebrados o la carne silvestre, lo que sugiere la posibilidad de una sustitución basada en el sabor. Entre los órdenes de mamíferos, los ungulados, los carnívoros, los primates y los roedores tuvieron un sabor similar, pero los pangolines (*Phataginus* sp. y *Smutsia gigantea*) tuvieron un sabor superior al de todos los órdenes excepto los roedores. Estos resultados sugieren que podría ser adecuado sustituir la carne silvestre por otros tipos de carne en función del sabor, excepto en el caso de los pangolines, que sólo pueden sustituirse adecuadamente por roedores.

## INTRODUCTION

Hunting wild animals for food is common in tropical regions, and it is driven mainly by subsistence and commercial demand (Ingram et al., 2021). However, hunting is associated with biodiversity loss, and offtake rates are largely unsustainable (IPBES, 2019; Ripple et al., 2016). Futher, hunting adversely affects ecosystem functioning and human well‐being by reducing tree recruitment, which decreases carbon storage, and by the loss of livelihoods and increasing the risk of zoonotic spillovers (Bello et al., 2015; Brashares et al., 2011; Friant et al., 2015).

Potential interventions to mitigate these impacts include introduction of alternative protein projects designed to reduce the consumption of particular species by offering substitute meats, such as fish, livestock, or captive‐bred wild species. These projects are based on the assumption that adoption of the substitute meat will decrease hunting pressure on species of conservation concern (Brittain et al., 2020; Secretariat of the Convention on Biological Diversity, 2011). A crucial part of such projects is the selection of appealing alternatives. However, selecting suitable substitutes is challenging because of the range of factors associated with wild meat consumption (Booker, 2019), including cultural significance, perceived health benefits, palatability, access, and cost (Chausson et al., 2019; Morsello et al., 2015; Nguyen et al., 2021; van Vliet & Mbazza, 2011; Wilkie et al., 2016).

Some of these factors (access and cost of substitutes) are external properties of the different meats and can be addressed through the intervention by improving access to markets or subsidizing the cost of substitutes, for example. However, other factors, particularly palatability, are inherent to the meats themselves, underscoring the need for robust assessments of preferences to determine the suitability of possible alternatives. Palatability (here, the perceived sensory qualities of meat flavor and texture) substantially influences human behavior across wild meat supply chains. Species considered more palatable are more likely to be hunted (Chaves et al., 2020), and the price per kilogram of wild meat is positively correlated with palatability (Emogor et al., 2025). Furthermore, consumers consider palatability when choosing what to eat, making it a key driver of meat consumption (Brittain et al., 2022; Tshikung et al., 2019). Despite its importance in guiding alternative protein interventions, to our knowledge, there has been no comprehensive assessment of the relative palatability of wild meat and alternatives to wild meat at site levels. Previous research, mainly across Africa, focused on a subset of the meats consumed in the study locations, which offers insights into which of the assessed subset are most and least preferred but not into preferences for other meats that may be consumed (Brittain et al., 2022; East et al., 2005; Friant et al., 2019; Ladele et al., 1996; Mwakatobe et al., 2012; Nguyen et al., 2021; Schenck et al., 2006).

We used data from three different stakeholder groups to assess the relative palatability of the main meats consumed in a biodiversity hotspot to inform the design of potential alternative protein interventions. Further, we examined the potential for substituting wild meat with other meat types based on palatability and explored differences in palatability of wild birds, mammals, and reptiles, and among different groups of mammals, which together represent the most hunted taxonomic class (Cawthorn & Hoffman, 2015; Ripple et al., 2016). Our results may help suggest when wild meat substitution is feasible and which alternatives are appropriate.

## METHODS

### Study location

From August to September 2022, we interviewed respondents in 15 communities around Nigeria's Cross River National Park (CRNP) (map of study area in Appendix S1). The park contains lowland and submontane forests and is in a biodiversity hotspot (Myers et al., 2000). It has two divisions: Oban (3000 km^2^) and Okwangwo (∼640 km^2^). Hunting poses the greatest direct threat to large‐bodied vertebrate species in the landscape (Agaldo et al., 2016) and is prohibited for certain groups (such as primates and pangolins) in and outside protected areas, but it is allowed for certain species when they are caught outside park boundaries (Endangered Species (Control of International Trade & Traffic) Act of Nigeria, 1985). We selected our study communities with stratified random and purposive sampling methods (details in Appendix S1).

### Respondent selection and data collection

We interviewed 570 adult respondents (18–80 years) on the palatability of solid animal‐derived protein (domestic meat, fish, invertebrate, and wild meat; hereafter meat). We separated respondents a priori into three categories: household, hunter, and vendor (i.e., sellers of wild meat in markets and restaurants). There were 190 respondents in each category. Ten respondents per category were interviewed in border communities (11 communities) and 20 respondents per category were interviewed in enclaves (referring to communities in park boundaries [*n* = 4]).

To select household respondents, we used systematic sampling. First, we counted the number of households in their respective communities to determine the sampling interval. Second, using each community's center (town hall or market square) as a reference point, we counted the houses along the streets and approached every fourth or fifth house, depending on the total number of houses we counted, to recruit participants. If a household declined to participate or household members were unavailable, we approached the neighboring household. We mostly surveyed adult females in households because, in this region, they perform most of the cooking (Ene‐Obong et al., 2018). We identified respondents in the hunter and vendor categories through community members and by visiting wild meat markets. Where we did not meet the target number of respondents per category in a given community, which was mostly the case for the hunter category, we increased the number in the next visited community to obtain equal number of respondents in each category.

All respondents gave free, prior, and informed consent for their participation. Our research protocol was approved by Cambridge University's Psychology Research Ethics Committee (application number: PRE.2021.071) (Appendix S2).

We assessed the palatability of meat from 96 animal species (or species groups; hereafter species) in two broad steps. First, using photos to aid identification, we sequentially asked respondents whether they had consumed the meat from the different species on our list, thus creating a respondent‐specific list of consumed species. The species shown to respondents reflected the main domestic and wild animals, fish, and invertebrates consumed as food by humans in the landscape. It was compiled during focus group sessions with 10–15 people in two communities in June 2021 (Appendix S1). We grouped certain species to ease scoring, such as hornbills, bats, and small birds. The final list contained 10 domestic meats, seven fishes, 10 invertebrates, and 69 wild meats (12 birds, 45 mammals, and 12 reptiles).

**FIGURE 1:**
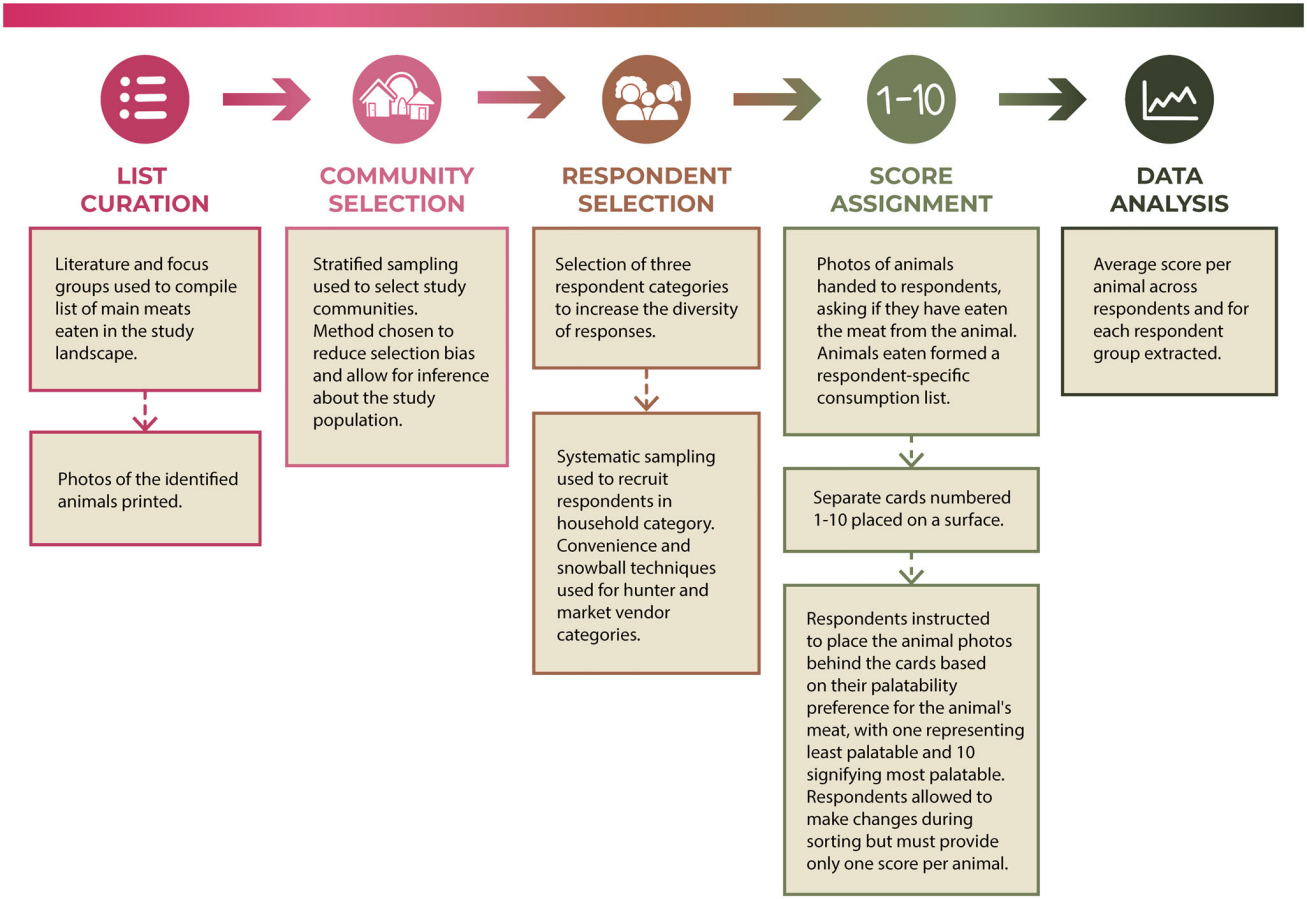
Summary of the protocol used to assess meat palatability. Graphics by Airi (Iris) Ryu.

In the second step, we arranged cards numbered 1–10 on a surface and asked respondents to place the photos of the selected species (i.e., only those the respondent chose in the first step) beneath a numbered card according to the respondent's preference for the meat of the species in question. Respondents scored each meat once: scores ranged from 1 (least palatable) to 10 (most palatable; protocol summary in Figure 1). We deployed the survey in English or Nigerian pidgin when respondents did not understand English (Appendix S1).

Although we did not explicitly shuffle the cards between respondents to control for order effects (i.e., the influence of the order in which items are presented on the outcome), each respondent first selected the animals they had consumed, which varied between respondents, and this varied the presentation order naturally. We did not use existing palatability assessment protocols, such as freelisting (participants mention their most and least preferred meats) (Quinlan, 2005) and taste experiments (participants taste cooked meat samples; Schenck et al., 2006) because we were interested in all the meats consumed in the landscape.

### Palatability and its correlates

To compare the palatability scores given by each respondent category, we first extracted the median palatability values per respondent category for each species. We used the median because of the skewed distribution of scores for some species. Next, we assessed the correlations (across species) in the median scores of our respondent categories: household versus hunter, household versus vendor, and hunter versus vendor. We excluded the vulture group because it was not scored by any hunter; thus, we had 95 species for the correlation tests.

We then investigated predictors of palatability with four simple linear regressions with Gaussian distributions. The first explored variations in palatability scores across respondent categories and meat types (category model). Similar to the first, the second (meat type model) looked at variation in overall palatability across meat types but restricting wild meat to species classed by International Union for Conservation of Nature (IUCN) as threatened (vulnerable, endangered, and critically endangered) because this group of species is usually the focus of conservation interventions. The third and fourth models examined variations in palatability of wild meats across taxonomic classes (class model) and of mammalian orders of wild meat (order model), respectively. In the category model, we modeled palatability (median palatability per species for each respondent category as the response variable) as a function of respondent category (household, hunter, and vendor) and meat type (domestic, fish, invertebrate, and wild). We fitted two category models: an additive model and an interaction model with an interaction term between these predictors. The interaction was based on an a priori expectation that hunters may be more likely than other respondents to find wild meat more palatable than other meat types. We used Akaike information criterion (AIC) to determine the most parsimonious of these two models. The response variable in the second model was the median palatability across respondent categories, which we fitted as a function of meat type, with wild meat restricted to threatened species only (*n* = 17 species).

In the class model, we focused on wild meat only and modeled palatability (median score per species averaged across all respondent categories) as a function of taxonomic class (bird, mammal, and reptile). Finally, in the order model with only wild mammals, we modeled palatability (median score averaged across all respondent categories) as a function of mammalian order (Artiodactyla, Carnivora, Pholidota, Primates, and Rodentia). We excluded Hyracoidea, Chiroptera, and Proboscidea because they each had only one record. Where there was a significant difference among predictor levels, we used Tukey's tests to compare the predictor levels with the R emmeans package (Lenth, 2023). The relationships between the categorical predictors and their respective response variables are in Appendix S3. We fitted the models in the stats package in R 4.2.2 (R Core Team, 2022) and used simulated residuals (Dunn & Smyth, 1996) to visually assess model fit by plotting residuals against each predictor variable (Appendix S3).

### Data availability

Data used in this article are available on Zenodo (https://doi.org/10.5281/zenodo.14897852).

## RESULTS

**FIGURE 2:**
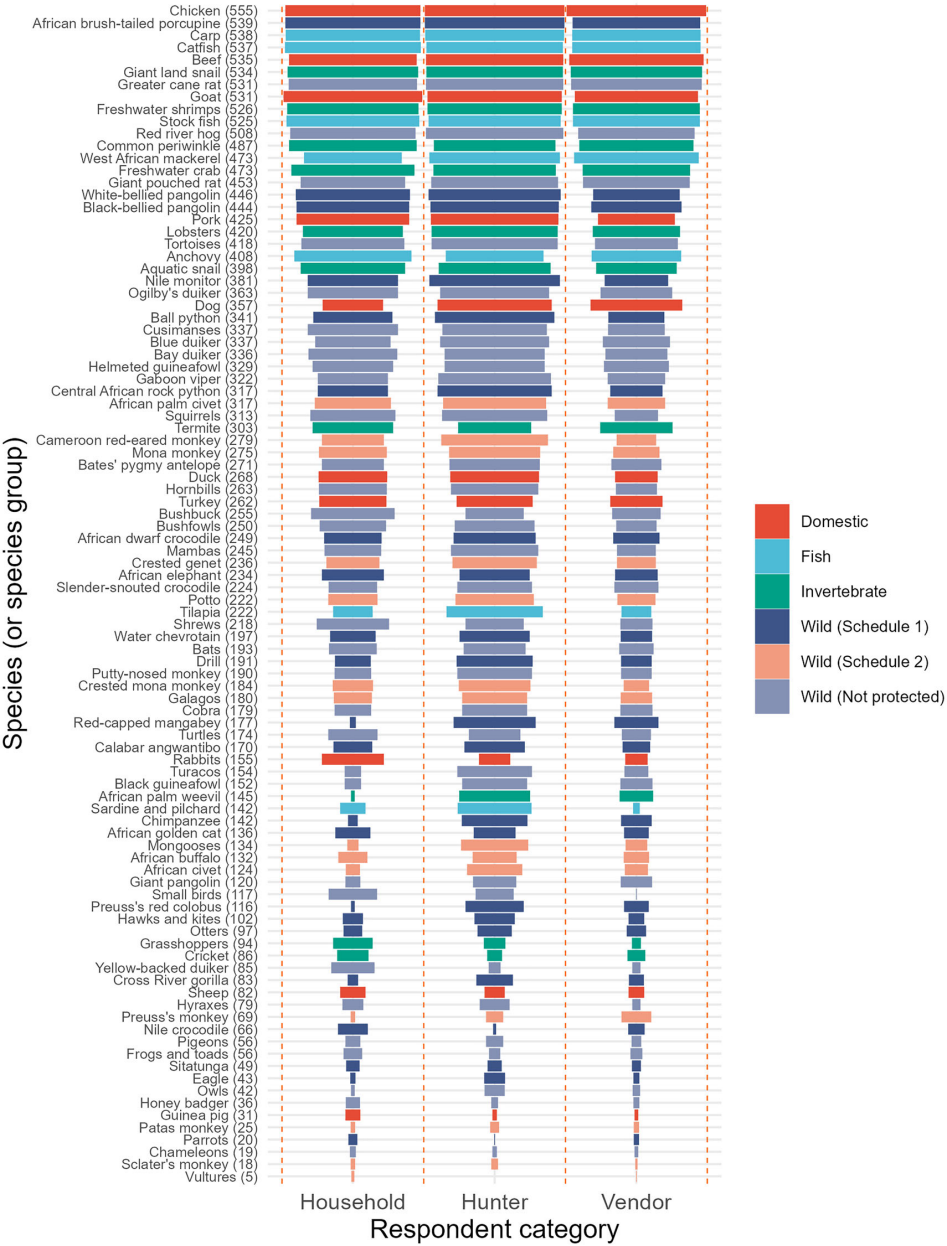
Proportion (length of bars) of respondents who scored each species (or species group) based on the palatability of their meat. (Schedule 1 are species whose hunting, capture, or trade are prohibited according to the *Endangered Species (Control of International Trade and Traffic) Act of 1985* [amended in 2016]. Schedule 2 are species whose hunting, capture, or trade are prohibited unless a license is issued granting permission (according to the act). Not protected are species or taxa not listed in the act. Values in parentheses are number of respondents across the three categories (household, hunter, and vendor) who scored each meat. In each category, 190 were surveyed (total 570).

**FIGURE 3:**
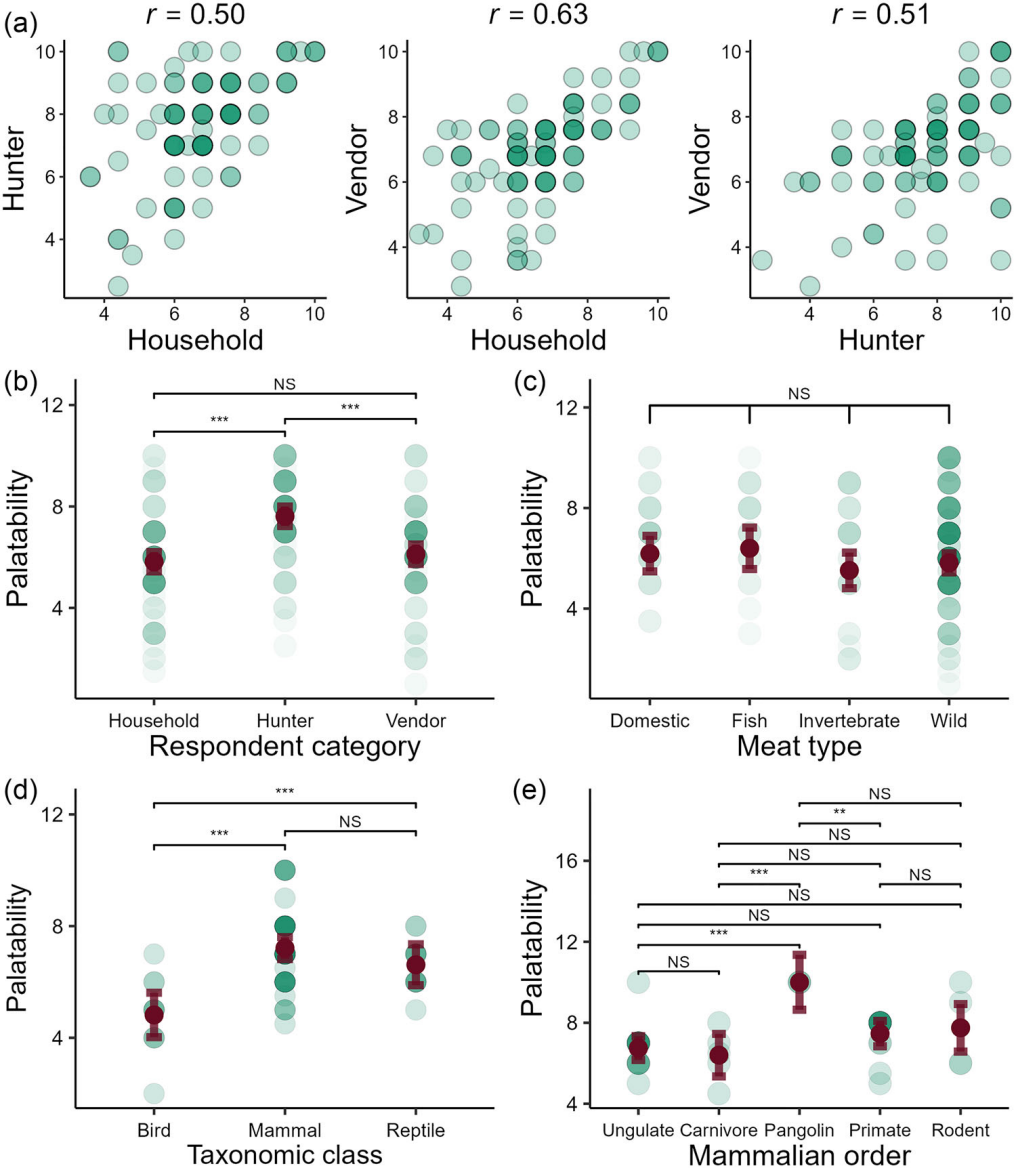
Correlations between meat palatability scored by (a) household respondents and hunters, household respondents and wild meat vendors, and hunters and vendors and differences in palatability across (b) respondent category, (c) meat type, (d) taxonomic class, and (e) taxonomic order. Circles are scores assigned to the meat of 96 species (excluding vultures in [a]). Darker points are overlapping species. Statistics in a‐c are the Pearson correlation for each category pair (each significant at *p* = 0.05). In (b–e) regression model predictions are shown with 95% confidence intervals (bars) (***, *p* < 0.001; **, *p* = 0.01; NS, nonsignificant). Data are median palatability scores of 570 respondents (190 household members, hunters, and vendors).

Respondents had a median age of 35 years (range 18–80). Women made up 57% of respondents (men = 43%). Across all categories, respondents consumed an average of 42.9 (SD 16.3) species (range 6–93). The mean figure for hunters was 52.8 (14.4) species (range 13–93), for household respondents 41.0 (13.2) (range 9–76), and for vendors 34.8 (15.7) species (range 6–85). The median values of the palatability scores across respondent categories and meat types ranged from 2 to 10. The scores assigned to domestic meat ranged from 4 to 9 (*n =* 11 meats, median = 7), fish were scored between 5 and 9 (*n =* 7, median = 7), invertebrates from 3 to 9 (*n =* 10, median = 7), and wild meat from 2 to 10 (*n =* 69, median of 6.5) (Appendix S4). African brush‐tailed porcupine (*Atherurus africanus*) was the species most scored and hence most eaten species in our sample (*n =* 539 different respondents). In contrast, vultures were eaten by only five of our respondents (Figure 2). Correlation coefficients indicated similarity in scores given by hunters, vendors, and other household members. All pairwise comparisons showed significant positive correlations (at *p* = 0.05) (Figure 3).

Model selection identified the additive category model as the most parsimonious. The model accounted for 16% of the variation in the response variable (median palatability for each species for each respondent category). Respondents who were hunters, on average, gave higher palatability ratings than respondents in the household and vendor categories (*β* = 1.79 [SE 0.26], *p* = <0.001 and *β* = 1.50 [0.26], *p* = <0.001 in post hoc test, respectively) (Figure 3b). The model showed no difference in palatability across meat types (Figure 3c). Similarly, the model comparing meat palatability of threatened wild species with other meat types did not show evidence of a difference (Table 1).

**TABLE 1:** Predicted variation in meat palatability scores across survey respondent categories and meat types (category model); in meat palatability of domestic meat, fish, invertebrates, and threatened wild species; in palatability across taxonomic classes; and in palatability across mammalian orders.

PREDICTOR	ESTIMATE (*β*)	SE	*t*	*p*
Category model				
Intercept	6.19	0.36	17.19	<0.001
Meat type: fish	0.21	0.51	0.42	0.68
Meat type: invertebrate	−0.67	0.46	−1.44	0.15
Meat type: wild meat	−0.37	0.35	−1.04	0.30
Respondent: hunter	1.79	0.26	6.91	<0.001
Respondent: vendor	0.30	0.26	1.15	0.25
Meat type model (threatened wild species only)				
Intercept	7.00	0.49	14.22	<0.001
Fish	0.21	0.76	0.28	0.78
Invertebrate	−0.45	0.70	−0.65	0.52
Wild meat	0.88	0.62	1.42	0.16
Class model				
Intercept	4.82	0.40	12.08	<0.001
Mammal	2.39	0.44	5.38	<0.001
Reptile	1.80	0.54	3.32	<0.001
Order model				
Intercept	6.75	0.29	23.30	<0.001
Carnivora	−0.35	0.59	−0.59	0.56
Pholidota	3.25	0.73	4.46	<0.001
Primates	0.71	0.42	1.68	0.10
Rodentia	1.00	0.65	1.54	0.13

The class model, which accounted for 28% of the variation in palatability (Table 1), showed that, on average, mammals were considered the most palatable class. Mammals and reptiles were more palatable than birds (*β* = 2.39 [SE 0.44], *p* < 0.001 and *β* = 1.80 [0.54], *p* < 0.001, respectively) (Figure 3d), but there was no evidence of a difference between mammals and reptiles. In the order model (explained 32% of variance) where Artiodactlya was the reference, only pangolins (Pholidota) had significantly different average palatability (*β* = 3.25 [0.73], *p* < 0.001) (Figure 3e). This was confirmed in post hoc tests (Appendix S4), where pangolins had higher palatability than carnivores (*β* = 3.6 [0.85], *p* = 0.001) and primates (*β* = 2.54 [0.74], *p* = 0.01) and palatability comparable with rodents (*β* = 2.25, [0.89], *p* = 0.10).

## DISCUSSION

### Correlations among respondents and differences across meat types

Respondents to our survey on meat palatability reported consuming an average of 43 species (approximately 70% of which are wild meat), underscoring the importance of wild meat for local communities. We found statistically significant correlations between the palatability scores provided by the three groups, suggesting a strong coherence in meat palatability preferences among people living in a rural area of southeast Nigeria. Although hunters had a significantly higher palatability rating overall (requiring further investigation), we found that overall respondents did not prefer a particular meat type (i.e., domestic, fish, invertebrate, or wild). Although respondents differed in the overall palatability values they reported, they did not differ in the relative values they assigned to different meat types (adding an interaction term weakened the category model).

Like other research on consumer preference for wild meat, there are important caveats to our work. Potential variations in palatability could arise from the diverse cooking methods used by respondents. However, we assumed that respondents would have consumed the various types of meat in different ways, allowing them to consider possible variations in palatability arising from cooking methods when scoring the meats. In addition, because we did not collect information on the rate of consumption of each meat, we could not test the hypothesis that a higher frequency of consumption positively affects consumer palatability preference (East et al., 2005; Schenck et al., 2006). Furthermore, because some of the species scored by respondents were protected, hence eating their meat is illegal, it is possible that some respondents may have misreported the species they have eaten. However, for those who admitted to eating protected species, it is unlikely that their palatability scores were misreported. Finally, our findings are limited to rural consumers, but we hope that future research will build on our work to explore meat palatability on a wider geographic scale.

Insofar as taste also influences wild meat consumption (Brittain et al., 2022; Nguyen et al., 2021), our finding of similarities in the palatability of the different meat types, including when we filtered wild meat to only threatened species, suggests the possibility of substituting wild meat with other types of meat in our study location. The comparability in palatability of fish, domestic, invertebrates, and wild contradicts previous work across Africa, including in our study location (Friant et al., 2019), showing a preference for one meat type over another. Researchers have reported a higher preference (based on palatability) for fish over wild meat in eastern Madagascar and northwestern Equatorial Guinea (East et al., 2005; Jenkins et al., 2011) and a high preference for wild meat over fish and domestic meat in Nigeria and Gabon (Brittain et al., 2022; Friant et al., 2019; Njiforti, 1996; Schenck et al., 2006). The rather different conclusions of these earlier studies may arise from the research methods adopted. The freelisting method used in Cameroon and Nigeria is susceptible to omissions and assumes that respondents list their most preferred item first and that the most frequently mentioned item across respondents is the most preferred, which may not always apply (Quinlan, 2005). Further, the higher preference for wild meat observed in Gabon was determined using a taste experiment with chicken and beef to represent domestic meat and African brush‐tailed porcupine and blue duiker (*Philantomba monticola*) to represent wild meat (Schenck et al., 2006). Importantly, the Gabon study accounted for variability that may arise from the preparation of the meat in question. However, the findings might only be relevant to the specific species examined.

### Palatability across wild meat groups

Our results showed that wild mammal meat is more palatable than meat from wild reptiles and birds. Given that hunters target the most palatable species, which also command them a higher price (Chaves et al., 2020; Emogor et al., 2025), this preference for mammals could partly explain their disproportionate contribution to wild meat offtake (Cawthorn & Hoffman, 2015; Ripple et al., 2016). We also found that the average palatability of pangolins was significantly higher than ungulates, carnivores, and primates (Figure 3e). The lack of statistical difference in the palatability of other pairs of orders further highlights the high palatability of pangolins. In addition, visual inspection of the palatability of domestic meat, fish, and invertebrates indicates a lower average palatability relative to pangolins, suggesting that rodents may be the only suitable replacement for pangolin meat. Two of the four African pangolin species exist in CRNP (*Phataginus tricuspis* and *P. tetradactyla*). The other species recorded in this study (*Smutsia gigantea*) occurs in neighboring parks in Cameroon and respondents in our study reported that it once existed in CRNP.

Other studies show that pangolin meat is highly palatable. Research from Cameroon, the Republic of the Congo, and Equatorial Guinea shows that pangolin meat is preferred because of its high palatability (Brittain et al., 2022; East et al., 2005; Nguyen et al., 2021; Swiacká et al., 2022). Our result on the palatability of pangolin meat relative to other meat types can inform interventions where pangolin poaching is primarily motivated by demand for meat (Emogor et al., 2023).

### Conservation implications

Our results suggest that the meat palatability preferences of three interrelated but different groups of people in southeast Nigeria are coherent, suggesting an opportunity to implement relatively cheaper alternative meat interventions because the same intervention may appeal to other groups of people. Our findings indicated that, based on palatability, domestic meat, fish, and invertebrates can serve as suitable substitutes for wild meat. Research in Ghana has shown that   low fish availability, resulting in increased fish prices, is linked to a simultaneous rise in the volume of wild meat sold in rural markets, suggesting that consumers consider wild meat as a viable alternative to fish and vice versa (Brashares et al., 2004).

However, such substitution may not apply to pangolins because they appear to have significantly higher palatability than the meat from domestic species, fish, invertebrates, and other mammals except rodents. This suggests that rodents, such as greater cane rat (*Thryonomys swinderianus*) and African brush‐tailed porcupine, with median palatability ratings of 9 and 10 in our study, respectively, could be the closest substitutes for pangolins. Both these species may be capable of withstanding greater levels of offtake because of their high fecundity and large litter size (Bennett et al., 2007; Cowlishaw et al., 2005; Okiwelu et al., 2010). Nonetheless, their use must comply with legal regulations in the intervention area. For instance, because hunting, trading, and consuming African brush‐tailed porcupines are illegal in Nigeria (Endangered Species (Control of International Trade & Traffic) Act of Nigeria, 1985), substituting pangolins with porcupines would conflict with legal regulations and may not be a viable alternative.

Despite the potential for wild meat substitution, interventions aimed at promoting alternative protein must address other factors associated with wild meat consumption, including the cost and availability of alternatives and the cultural significance of wild meat (Brittain et al., 2022; Chausson et al., 2019; Wilkie et al., 2016). Such interventions should also work with communities to identify economically viable alternatives, develop contextually relevant food preparation skills (Chaves et al., 2017), and ensure that the supply of alternative protein matches the historical supply of wild meat (Wilkie et al., 2016).

## AUTHOR CONTRIBUTIONS

CONCEPTUALISATION. Idea formulation: Charles A. Emogor (lead) and Andrew Balmford. Brainstorming: Charles A. Emogor (lead) and Andrew Balmford. Concept development: Isa B. Ebri, Benedict A. Atsu, Dominic S. Ogu, Omini B. Iferi, and Andrew Balmford. DATA ACQUISITION. Data collection: Isa B. Ebri, Benedict A. Atsu, and Dominic S. Ogu. Research operations coordination: Charles A. Emogor. Data contributors: 570 anonymous volunteers. DATA PROCESSING AND ANALYSIS. Data cleaning and preprocessing: Charles A. Emogor. Data analyses: Charles A. Emogor. VISUALISATION. Analysis tools: R and R studio (ggplot2, tidyverse, ggsignif, and performance) and QGIS. Illustration: Airi (Iris) Ryu (Figure 1). Map: Charles A. Emogor. MANUSCRIPT. Original draft: Charles A. Emogor. Internal review: Isa B. Ebri, Benedict A. Atsu, Dominic S. Ogu, Omini B. Iferi, Andrew Balmford (lead). External review: J. P. G. Jones and two anonymous reviewers. Revision: Charles A. Emogor. LOGISTICS. Research permit: Charles A. Emogor, Wildlife Conservation Society, Nigeria (facilitated approval). Ethics protocol: Charles A. Emogor and two anonymous reviewers. Risk assessment: Charles A. Emogor and Sylviane Moss (reviewer). LOCATION. Location authorization: Nigeria National Park Service. PROJECT MANAGEMENT. Training of data collectors: Charles A. Emogor. Funding management: Charles A. Emogor. Data management: Charles A. Emogor. Supervision: Andrew Balmford. Quality control: Charles A. Emogor. The contributions follow the Extended Research Credits framework.

## CONFLICT OF INTEREST STATEMENT

The authors are affiliated with Pangolin Protection Network (https://pangolino.org/), a conservation nonprofit promoting community based interventions to reduce pangolin decline in Nigeria.

## Supporting information



Supporting Information
