# Supplementary material for: Assessing the palatability of different meats consumed in a biodiversity hotspot to inform alternative protein interventions
Source: Conserv Biol. 2025 Apr 22;39(4):e70026. doi: 10.1111/cobi.70026 (PMC12309634; doi:10.1111/cobi.70026)
Supplement: Supplementary file 1 — Supporting Information [file COBI-39-e70026-s001.docx]

SUPPLEMENTARY MATERIAL

Assessing the palatability of different meats consumed in a biodiversity hotspot to inform alternative protein interventions

Charles A. Emogor^1,2,3🖂^, Isa B. Ebri^3^, Benedict A. Atsu^3^, Dominic S. Ogu^3^, Omini B. Iferi^3^, and Andrew Balmford^1^

^1^ Conservation Science Group, Department of Zoology, University of Cambridge, UK

^2^ Wildlife Conservation Society, Calabar, Nigeria

^3^ Pangolin Protection Network (Pangolino), Calabar, Nigeria

^🖂^ Charles A. Emogor; [emogorcharles@gmail.com](mailto:emogorcharles@gmail.com)

Appendix S1

Supplementary Methods

Community selection

We used a combination of stratified random and purposive sampling to select our focal communities, with the selection strategy varying between the two divisions of Cross River National Park (CRNP). We used the stratified random technique in Okwangwo by first dividing the landscape into four geographic quadrants before randomly selecting communities: two from the southeast, southwest, and northwest and one from the northeast (due to logistical constraints). Three of these communities were within park boundaries (enclaves). In Oban, applied purposive sampling by simpling selecting communities that have previously conducted wild meat research, i.e., monitoring wild meat hunting (n = 2 communities), market sales of wild meat (n = 2), and household meat consumption (n = 4). The two hunting communities border each other in the northwestern part of Oban and were selected for their relative ease of recruiting hunters – our work with hunters in these communities was facilitated by a wild meat study conducted about a decade ago (Vath, 2014). One of the wild meat trade communities is located on the northern borders of Oban, while the other lies on the southern border (approximately 80 km apart). The four meat consumption communities were randomly selected, with one community chosen from each of the four geographic quadrants of the division; one of these communities was an enclave (Figure 1).


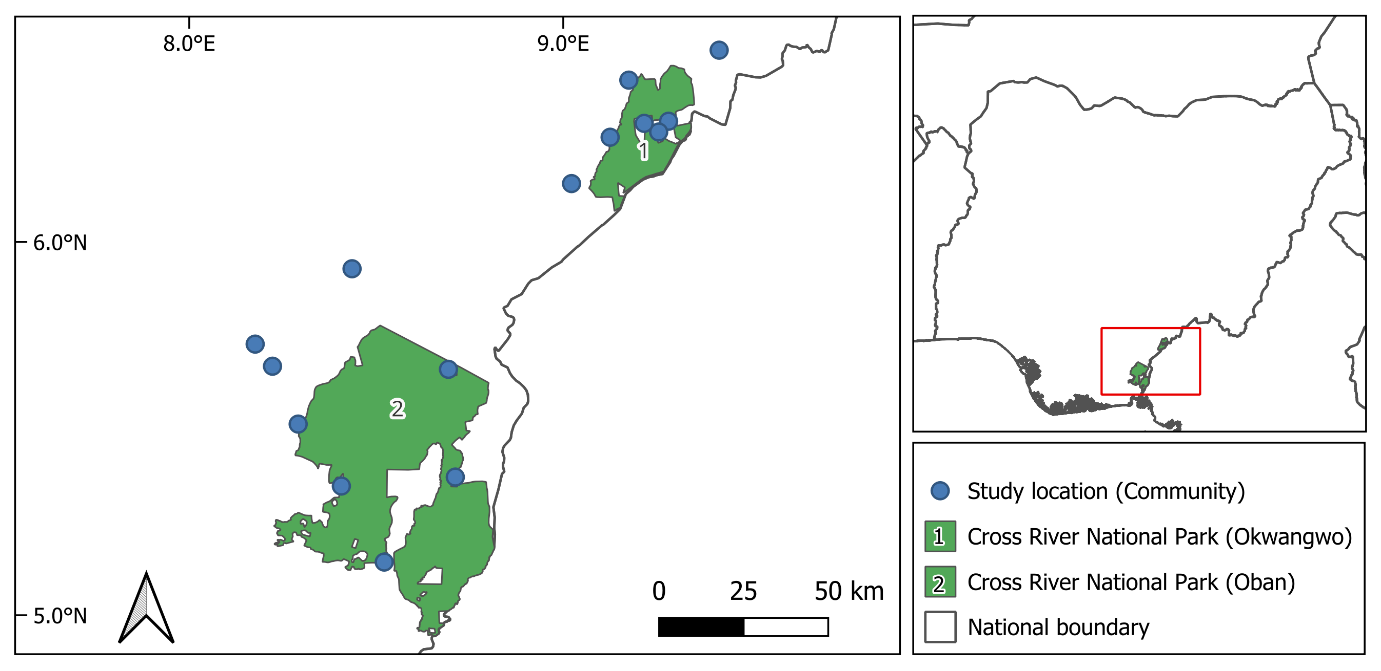


Figure 1: Approximate locations of our study communities surveyed relative to Nigeria’s Cross River National Park (red rectangle). Community names are not provided to maintain anonymity.

Focus group discussions

We asked for volunteers via the community leaders and worked with the first 10-15 people who showed up. We asked them to independently list the animals whose meat they and others in the community consume for food. When they had all done so, we compiled the lists and discussed the aggregated list with all participants, allowing us to consolidate the individual lists. Next, we matched the consolidated list against the species recorded in our ongoing work with hunters and vendors in the landscape, adding more species to the final list based on our survey records.

Interview questions (pidgin translations are provided in parentheses)

Question 1: What is the name of the community?

*Note: The list of communities was determined before the survey; see Methods.*

Question 2: What is the category of the respondent?

*Note: The categories were determined before the survey; see Methods*.

Question 3: What is your sex (you be man or woman)?

Question 4: What is your year of birth (watin be the year wey them born you)?

*Note: We only interviewed people from 18 years.*

Question 5: Which of these animals have you eaten meat from (which of these animal wey you don chop im meat)?

*Note:* H*ere we handed the photos of the different species to participants to sort the animals whose meat they had eaten.* The list of species are presented in Table S1.

Question 6: Rank the meat from these animals [*specifically the animals they reportedly ate*] on a scale of 1-10, with 10 being most palatable and 1 being least palatable (give the meat from the animals wey you don chop numbers based on how much you like the taste, smell, and toughness of the meat. Give 1 to the meat were you no too like and 10 to meat wey you like well well).

*Note: Here we provided cards numbered 1-10 to aid scoring. See Methods.*

References

Vath, C. L. (2014). Social and economic dimensions of the bushmeat trade in cross river state nigeria: An ethno-biological approach to conservation.

Appendix S2

Participant information sheet


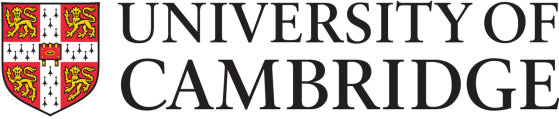


**Department of Zoology**

**Address:** Downing Street, Cambridge, CB2 3EJ, United Kingdom

**Email:** [reception@zoo.cam.ac.uk](mailto:reception@zoo.cam.ac.uk?subject=Message%20from%20Website)

**Tel:** +44 (0)1223 336600

**Understanding the Role of Wildmeat in the Livelihoods of Communities Surrounding the Cross River National Park, Nigeria**

*Before you decide to take part in this study, it is important for you to understand why the research is being done and what it will involve. Please take time to read the following information carefully and discuss it with others if you wish. A member of the team can be contacted if there is anything that is not clear or if you would like more information. Take time to decide whether or not you wish to take part.*

**Purpose of the study**

Wild meat is an important resource in the livelihoods of many rural communities in large parts of Africa. However, the population of most animals harvested for food is declining. Their decline could mean possible loss of livelihoods for people who depend on them for food and income. We want to understand the role wild meat plays in the livelihoods of people living immediately around the CRNP. Our study will inform future conservation actions to reduce species decline and to preserve the livelihoods of people who depend on wild meat for food and income.

**Why have I been chosen?**

You have been selected to partake in this study because you live in one of the communities surrounding the Cross River National Park (CRNP) and hunt and sell wild animal.

**Do I have to take part?**

Your participation is entirely voluntary, and if you decide to take part, you are free to withdraw at any time.

**What will happen to me if I take part?**

We will ask you some questions about wild meat (“bushmeat”), hunting, and use in your community and will record your responses with a mobile device. For hunters, we will collect data on the animals that you catch after every hunting trip and will sometimes weigh the carcass in your presence. For other groups, we will only ask you about the type of meat you have eaten and also how much you like the taste of the different meats you have eaten. The interview will last for 20-30 mins.

**Are there possible disadvantages and/or risks in taking part?**

The information you will provide might threaten your livelihoods as this will be used to target enforcement (such as anti-poaching patrols) to reduce wild animal decline.

**What are the possible benefits of taking part?**

Your participation will provide useful information needed to conserve wild animals and protect the livelihoods of people around forests and other places where wild animals live. You will also receive a thank you souvenir at the end of the interview.

**Will my taking part in this project be kept confidential?**

All data will be identified only by a code, with personal details kept in a locked file or secure computer with access only by the immediate research team. Please follow the link below for general information about how the University of Cambridge uses personal data: <https://www.information-compliance.admin.cam.ac.uk/data-protection/research-participant-data>.

**What will happen to the results of the research project?**

Results will be presented at conferences and written up in journals.  Results are normally presented in terms of groups of individuals. If any individual data are presented, the data will be totally anonymous, without any means of identifying the individuals involved. We will likely use this data for subsequent research.

**Who is organising and funding the research?**

This study is partly funded by the Gates Cambridge Trust (University of Cambridge).

**Ethical** **review of the study**

This project has been reviewed by the University of Cambridge Psychology Research Ethics Committee. Additionally, a comprehensive risk assessment has been carried out for the researchers including assistants and participants who volunteer to take part in this study.

**Contact for further information**

If you decide to take part, please contact Charles Emogor at [cae37@cam.ac.uk](mailto:cae37@cam.ac.uk) or call 081XXXXXXX. Thank you.

Consent Form


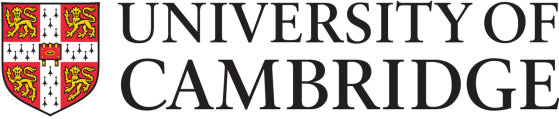


**Department of Zoology**

**Address:** Downing Street, Cambridge, CB2 3EJ, United Kingdom

**Contact:** [reception@zoo.cam.ac.uk](mailto:reception@zoo.cam.ac.uk?subject=Message%20from%20Website) | +44 (0)1223 336600

**Consent Form**: **Understanding the Role of Wildmeat in the Livelihoods of Communities Surrounding the Cross River National Park, Nigeria**

Please tick the boxes below as appropriate.

- *I confirm that I have read and understood the Participant Information Sheet*
- *I have had the opportunity to ask questions and had them answered*
- *I understand that all personal information will remain confidential and that all efforts will be made to ensure I cannot be identified (except as might be required by law)*
- *I understand that the data will be stored anonymously and securely, and may be used for future research*
- *I understand that my participation is voluntary and that I am free to withdraw at any time without giving a reason*
- *I agree to take part in this study*

Location (including community name): _______________________________________

Participant’s signature: _______________________ Date: ____________________

Investigator’s name: ____________________________________ Signature: _________________

Date: ______________________

Appendix S3: Figures


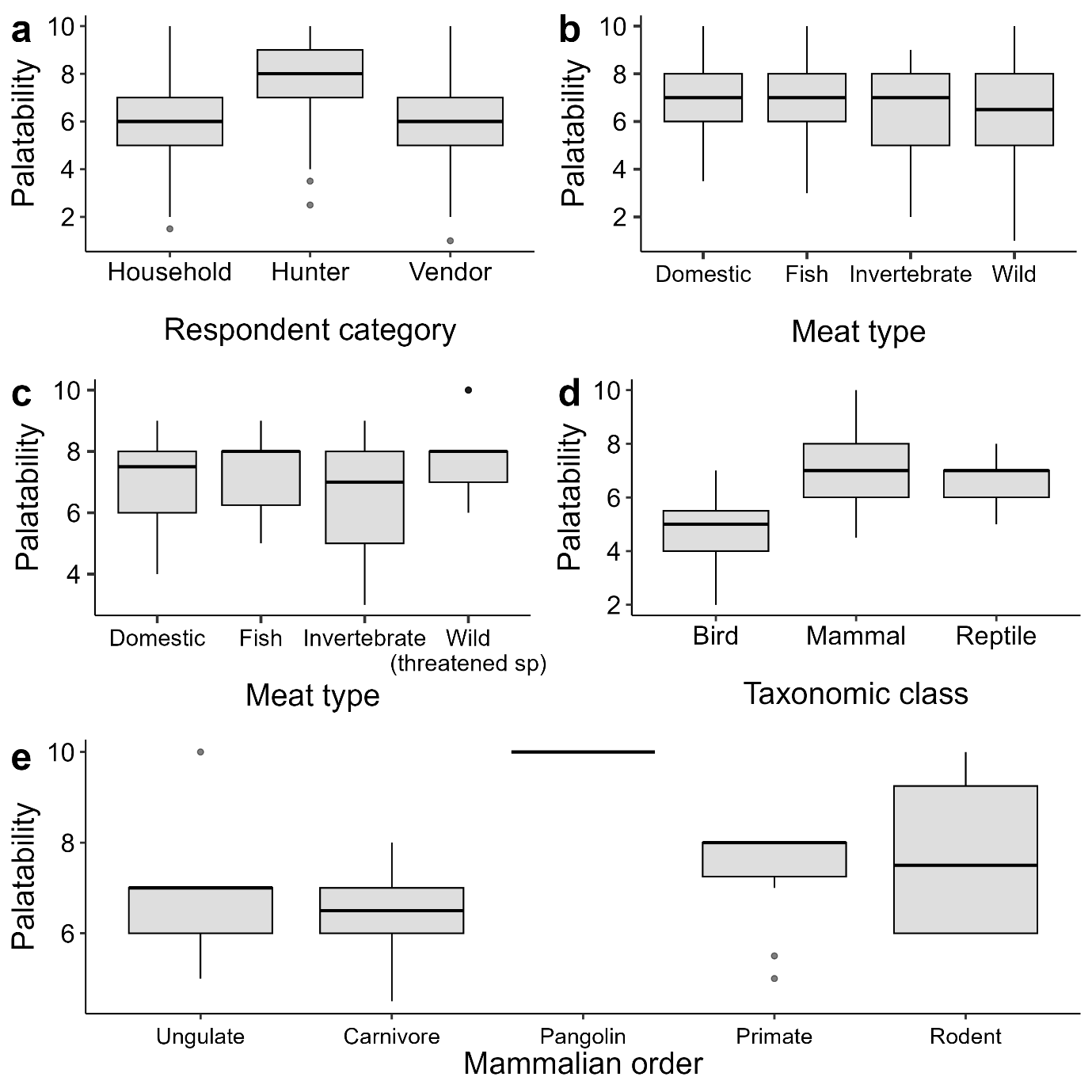


**Figure S1**: Relationships between the response variables and predictors used in the models. Respondent category and meat type (a and b) were used in the category model, where the response was the median palatability per animal for each respondent category. In c, we restricted wild meat to threatened species (n = 17 species) based on International Union for Conservation of Nature (IUCN) and averaged palatability across respondent categories. Taxonomic class (d) was used to model the variation in palatability across taxonomic class (wild meat only) with the response being the median score per animal averaged across all respondent categories (class model). In modelling the variation in palatability across mammalian order (order model; e), we used the median score per animal averaged across all respondent categories as the response. The grey boxes represent the interquartile range of the data (between the first and the third quartiles), with the median number of animals captured shown by the thick horizontal line in each box. Whiskers show the minimum and maximum values of the data for each community and outliers are represented by the dots. The plot was made using built-in functions in R. The data represents the median palatability of the meat from 96 animals scored by 570 respondents (190 household members, hunters, and wild meat vendors).


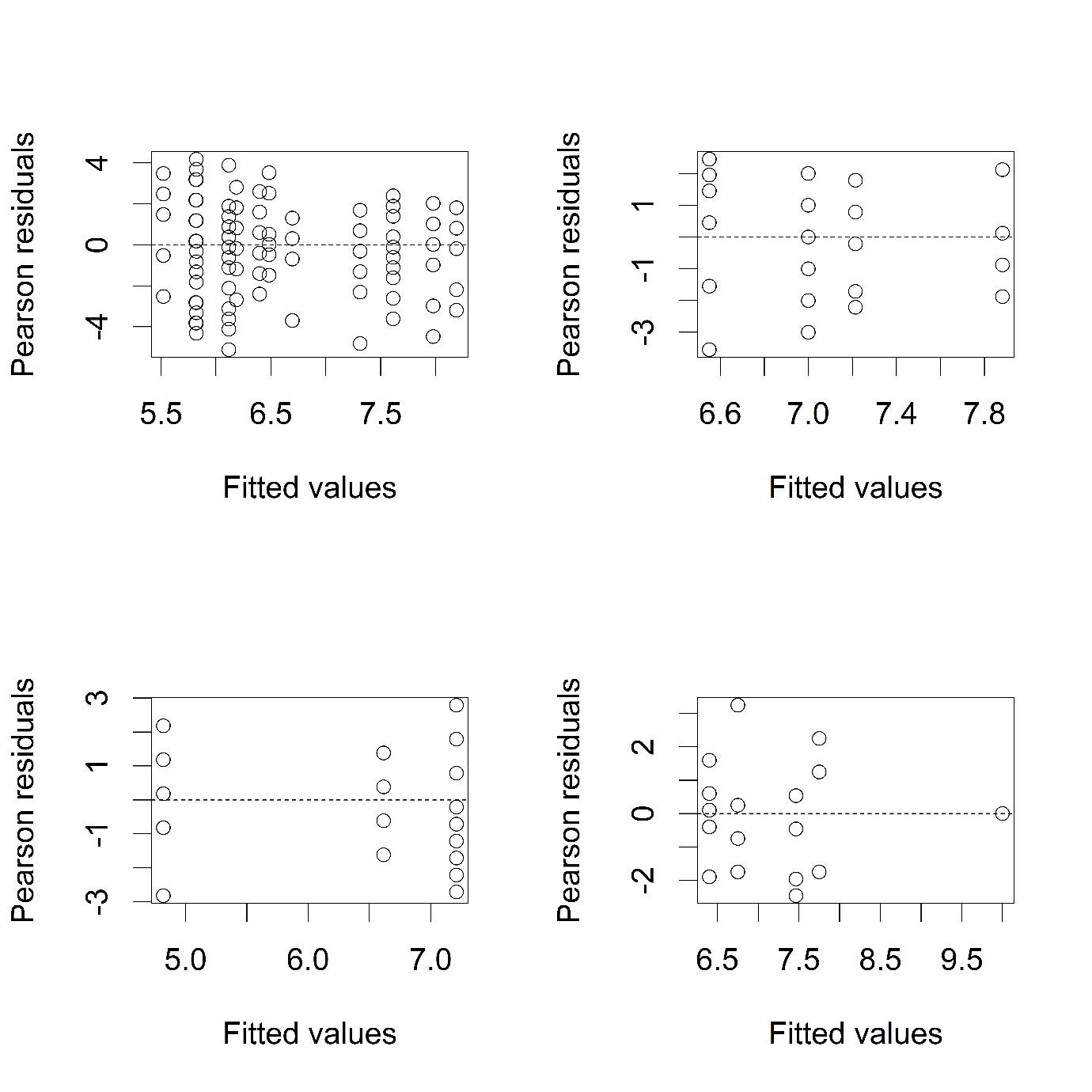


**Figure S2**: Pearson residual from the category (top left), meat type (top right), class (bottom left), and order (bottom right) models. The fairly random distribution of the Pearson residuals around 0, shown by the dotted horizontal line, suggests a good model fit. Plot made using built-in functions in R. The data represents the median palatability of the meat from 96 animals scored by 570 respondents (190 household members, hunters, and wild meat vendors).

Appendix S4: Tables

**Table S1**: Median palatability scores of 96 animals provided by 570 respondents in three categories (household, subsistence hunter, and wild meat vendor) in south-east Nigeria. Scores were sorted by meat type and then by taxonomic class for wild meat. Palatability surveys were conducted in August-September 2022.

| Species (Taxonomy) | Palatability score | | | |
| --- | --- | --- | --- | --- |
|  | Overall | Household | Hunter | Vendor |
| **Domestic meat** | |  |  |  |
| Cattle (*Bos taurus*) | 8 | 8 | 7 | 7 |
| Chicken (*Gallus gallus*) | 9 | 9 | 9 | 10 |
| Dog (*Canis faimiliaris*) | 9 | 7 | 10 | 9 |
| Duck (Anatidae) | 5 | 6 | 5 | 5 |
| Goat (*Capra hircus*) | 8 | 8 | 8 | 7 |
| Guinea pig (*Cavia porcellus*) | 4 | 3.5 | 3.5 | 5 |
| Pig (*Sus domestica*) | 8 | 6 | 8 | 7 |
| Rabbit (Leporidae) | 6 | 5 | 8 | 6.5 |
| Sheep (*Ovis aries*) | 6 | 6 | 7 | 6 |
| Turkey (*Meleagris* spp.) | 7 | 6 | 7 | 7 |
|  |  |  |  |  |
| **Fish** | |  |  |  |
| Anchovy (Engraulidae) | 5.5 | 6 | 6 | 3 |
| Carp (Cyprinidae) | 8 | 9 | 9 | 7 |
| Catfish (Siluriformes) | 9 | 9 | 10 | 8 |
| Sardine and pilchard (Alosidae) | 5 | 4 | 5 | 7 |
| Stock fish | 7 | 7 | 8 | 6 |
| Tilapia (*Oreochromis* spp.) | 8 | 5 | 9 | 7 |
| Mackerel (*Scomberomorus* spp.) | 8 | 8 | 8 | 8 |
|  |  |  |  |  |
| Invertebrate | |  |  |  |
| African palm weevil (*Rhynchophorus phoenicis*) | 8 | 5 | 8 | 2 |
| Aquatic snail (Melanopsidae) | 7 | 7 | 6 | 7 |
| Common periwinkle (Littorinidae) | 7 | 7 | 7 | 7 |
| Cricket (Grylloidea) | 3 | 3 | 2.5 | 2 |
| Freshwater crab (Potamidae) | 5 | 5 | 5 | 6 |
| Freshwater shrimp (Pleocyemata) | 9 | 9 | 9 | 9 |
| Giant African land snail (Achatinidae) | 8.5 | 8 | 9 | 9 |
| Grasshopper (Acrididea) | 5 | 5 | 8 | 3 |
| Lobster (Nephropidae) | 8 | 7 | 9 | 8 |
| Termite (Isoptera) | 5 | 5 | 7 | 2 |
|  |  |  |  |  |
| **Wild meat** | |  |  |  |
| Bird | |  |  |  |
| Black guineafowl (*Agelastes niger*) | 6 | 5 | 7 | 6 |
| Bush fowl (Numididae**)** | 6 | 6 | 8 | 5 |
| Eagle (Accipitridae) | 5 | 2 | 6 | 6 |
| Hawk and kite (Accipitridae) | 4 | 3 | 4 | 5 |
| Helmeted guineafowl (*Numida meleagris*) | 7 | 7 | 8 | 7 |
| Hornbill (Bucerotidae) | 5 | 5 | 5 | 6 |
| Owl (Strigiformes) | 5 | 2 | 6 | 3 |
| Parrot (Psittaciformes) | 4 | 3 | 10 | 6 |
| Pigeon (Columbiformes) | 5 | 4.5 | 8 | 5 |
| Small bird | 4 | 3 | 4 | 1 |
| Turaco (Musophagidae) | 7 | 2.5 | 8 | 7 |
| Vulture (Gypaetinae and Aegypiinae) | 2 | 1.5 | - | 3 |
|  |  |  |  |  |
| Mammal | |  |  |  |
| African brush-tailed porcupine (*Atherurus africanus*) | 10 | 10 | 10 | 10 |
| African buffalo (*Syncerus caffer*) | 6 | 7 | 6 | 5 |
| African civet (*Civettictis civetta*) | 6.5 | 5 | 8 | 5 |
| African elephant (*Loxondonta africana*) | 8 | 7 | 8 | 8 |
| African golden cat (*Caracal aurata*) | 6 | 5 | 7 | 6 |
| African palm civet (*Nandinia binotata*) | 8 | 7 | 9 | 8 |
| Bates’ pygmy antelope (*Nesotragus batesi*) | 7 | 7 | 8 | 7 |
| Bats (Chiroptera) | 5 | 5 | 5 | 2.5 |
| Bay duiker (*Cephalophus dorsalis*) | 7 | 6 | 7 | 7 |
| Black-bellied pangolin (*Phataginus tetradactyla*) | 10 | 10 | 10 | 10 |
| Blue duiker (*Philantomba monticola*) | 7 | 6 | 7 | 6.5 |
| Bushbuck (*Tragelaphus scriptus*) | 6 | 5.5 | 7 | 6 |
| Calabar angwantibo (*Arctocebus calabarensis*) | 5 | 5 | 4 | 5 |
| Cameroon red-eared monkey (*Cercopithecus* *erythrotis*) | 8 | 7 | 9 | 8 |
| Chimpanzee (*Pan troglodytes*) | 8 | 4 | 9 | 7 |
| Crested genet (*Genetta cristata*) | 7 | 6 | 7 | 6 |
| Crested mona monkey (*Cercopithecus pogonias*) | 8 | 7 | 8 | 7.5 |
| Cross River gorilla (*Gorilla gorilla diehli*) | 7 | 6 | 9 | 5 |
| Cusimanse (*Crossarchus* spp*.)* | 7 | 7 | 8 | 7 |
| Drill (*Mandrillus leucophaeus*) | 8 | 8 | 9 | 7 |
| Galago (Galagidae) | 5.5 | 5 | 5 | 6.5 |
| Giant pangolin (*Smutsia gigantea*) | 10 | 9.5 | 10 | 10 |
| Giant pouched rat (*Cricetomys* spp.) | 7 | 6 | 7 | 6 |
| Greater cane rat (*Thryonomys swinderianus*) | 9 | 9 | 10 | 8 |
| Honey badger (*Mellivora capensis*) | 4.5 | 5 | 7 | 4 |
| Hyrax (Hyracoidea) | 6 | 6 | 7.5 | 5 |
| Mona monkey (*Cercopithecus mona*) | 8 | 6 | 9 | 7 |
| Mongoose (Herpestidae) | 7 | 5 | 8 | 6 |
| Ogilby's duiker (*Cephalophus ogilbyi*) | 6 | 6 | 7 | 6 |
| Otters (Lutrinae) | 6 | 4 | 7.5 | 5.5 |
| Patas monkey (*Erythrocebus patas*) | 8 | 6 | 10 | 4 |
| Milne-Edwards’s potto (*Perodicticus edwardsi*) | 6 | 5 | 6 | 5 |
| Preuss's red colobus (*Piliocolobus preussi*) | 8 | 3 | 8 | 7 |
| Preuss's monkey (*Allochrocebus preussi*) | 7 | 3 | 9 | 6 |
| Putty-nosed monkey (*Cercopithecus nictitans*) | 8 | 6 | 9 | 7 |
| Red river hog (Potamochoerus porcus) | 10 | 9 | 10 | 8 |
| Red-capped mangabey (*Cercocebus torquatus*) | 8 | 5 | 9 | 8 |
| Sclater’s monkey (*Cercopithecus sclateri*) | 8 | 3 | 10 | 4 |
| Shrew (Soricidae) | 6 | 6 | 7 | 6 |
| Sitatunga (*Tragelaphus spekii*) | 7 | 5 | 7 | 7 |
| Squirrel (Sciuridae) | 6 | 5 | 7 | 6 |
| Water chevrotain (*Hyemoschus aquaticus*) | 7 | 6 | 8 | 7 |
| White-bellied pangolin (*Phataginus tricuspis*) | 10 | 10 | 10 | 10 |
| Yellow-backed duiker (*Cephalophus silvicultor*) | 5 | 3 | 6.5 | 6 |
|  |  |  |  |  |
| Reptile | |  |  |  |
| African dwarf crocodile (*Osteolaemus tetraspis*) | 7 | 6 | 8 | 6.5 |
| Slender-snouted crocodile (*Mecistops cataphractus*) | 8 | 7 | 9 | 6 |
| Ball python (*Python regius*) | 7 | 7 | 9 | 6 |
| Central African rock python (*Python sebae*) | 7 | 6 | 9 | 6 |
| Chameleon (Chamaeleonidae) | 5 | 5.5 | 10 | 2 |
| Cobras (Elapidae) | 6 | 6 | 7 | 5 |
| Frog and toad (Anura) | 6 | 5 | 7 | 6.5 |
| Gaboon viper (*Bitis gabonica*) | 7 | 6 | 8 | 6 |
| Mamba (*Dendroaspis* spp.) | 6 | 6 | 7 | 6 |
| Nile crocodile (*Crocodylus niloticus*) | 6 | 5 | 9.5 | 6.5 |
| Nile monitor (*Varanus niloticus*) | 8 | 7 | 8 | 8 |
| Tortoise (Testudinidae) | 7 | 7 | 8 | 5 |
| Turtle (Testudines) | 6 | 5 | 7 | 5 |

**Table S2**: Coefficient of pairwise comparisons of levels within categorical predictors used to predict variation in palatability scores across respondent categories and meat types (category model), variation in palatability across taxonomic classes (restricted to wild meat; class model), and variation in palatability across mammalian orders (order model). We did not provide the coefficients of the meat type model as no predictor showed evidence of an effect.

| Pair | Estimate (*β*) | Std. Error (*SE*) | *t* ratio | *P* value |
| --- | --- | --- | --- | --- |
| *Category model* | | | | |
| Domestic meat - Fish | -0.21 | 0.51 | -0.42 | 0.98 |
| Domestic meat - Invertebrate | 0.67 | 0.46 | 1.44 | 0.48 |
| Domestic meat - Wild meat | 0.37 | 0.35 | 1.04 | 0.72 |
| Fish - Invertebrate | 0.88 | 0.51 | 1.72 | 0.31 |
| Fish - Wild meat | 0.58 | 0.41 | 1.41 | 0.50 |
| Invertebrate - Wild meat | -0.30 | 0.35 | -0.86 | 0.83 |
| Household - Hunter | -1.79 | 0.26 | -6.91 | < 0.0001 |
| Household - Vendor | -0.30 | 0.26 | -1.15 | 0.49 |
| Hunter - Vendor | 1.50 | 0.26 | 5.77 | < .0001 |
|  |  |  |  |  |
| *Class model* | | | | |
| Bird - Mammal | -2.39 | 0.45 | -5.38 | < 0.001 |
| Bird - Reptile | -1.80 | 0.54 | -3.32 | 0.0001 |
| Mammal - Reptile | 0.60 | 0.42 | 1.43 | 0.33 |
|  |  |  |  |  |
| *Order model* | |  | | |
| Artiodactyla - Carnivora | 0.35 | 0.59 | 0.59 | 0.98 |
| Artiodactyla - Pholidota | -3.25 | 0.73 | -4.46 | < 0.001 |
| Artiodactyla - Primates | -0.71 | 0.42 | -1.68 | 0.46 |
| Artiodactyla - Rodentia | -1 | 0.65 | -1.54 | 0.54 |
| Carnivora - Pholidota | -3.6 | 0.85 | -4.25 | 0.001 |
| Carnivora - Primates | -1.06 | 0.60 | -1.76 | 0.41 |
| Carnivora - Rodentia | -1.35 | 0.78 | -1.74 | 0.42 |
| Pholidota - Primates | 2.54 | 0.74 | 3.44 | 0.01 |
| Pholidota - Rodentia | 2.25 | 0.89 | 2.54 | 0.10 |
| Primates - Rodentia | -0.29 | 0.66 | -0.44 | 0.99 |

Appendix S5: **Extended Research Credits**

**Funding**

Author salary: Bill & Melinda Gates Foundation (OPP1144; C.A.E.), Pangolin Protection Network (I.B.E., B.A.A., D.S.O., O.B.I.) and University of Cambridge (A.B.). Data: National Geographic Society Early Career Grant (EC-83678R-21). Open access publishing: University of Cambridge

Conceptualization

Idea formulation: C.A.E (lead) and A.B. Brainstorming: C.A.E. (lead) and A.B. Concept development: C.A.E. (lead), I.B.E., B.A.A., D.S.O., O.B.I., A.B. and D.J. Ingram (provided feedback on the questionnaire).

Data acquisition

Data collection: I.B.E., B.A.A., D.S.O. Research operations coordination: C.A.E. Data contributor: 570 anonymous volunteers.

Data processing and analysis

Cleaning and preprocessing: C.A.E. Analysis: C.A.E. Tools (software): R and R studio (ggplot2, tidyverse, ggsignif, performance) and QGIS

Visualization

Illustration: Airi (Iris) Ryu (Figure 1). Map: C.A.E.

Manuscript

Original draft: C.A.E. Internal review: I.B.E., B.A.A., D.S.O., O.B.I., A.B (lead). External review: J.P.G Jones and two anonymous reviewers. Revision: C.A.E.

Logistics

Research permit: C.A.E., Wildlife Conservation Society, Nigeria (facilitated approval). Ethics: C.A.E. and two anonymous reviewers. Risk assessment: C.A.E. and Sylviane Moss (reviewer).

Location

Location authorization: Nigeria National Park Service

Project management

Training: C.A.E. Funding management: C.A.E. Data management: C.A.E. Supervision: A.B. Quality control: C.A.E.
